# Albumin-Like Proteins Are Critical Regulators of Vascular Redox Signaling

**DOI:** 10.1155/2013/628615

**Published:** 2013-02-05

**Authors:** Kenneth S. Ramos, Vilius Stribinskis, Marlene C. Steffen, Adrian Nanez, Diego Montoya-Durango, Qiang He

**Affiliations:** Department of Biochemistry and Molecular Biology, University of Louisville School of Medicine, 580 South Preston Street, Louisville, KY 40202, USA

## Abstract

This laboratory previously identified an albumin-like protein (denoted as p70) as a component of the macromolecular complex assembled within the 5′-regulatory region of redox-sensitive genes in vascular smooth muscle cells (vSMCs). Here we show that p70 is present in the cytosolic and nuclear compartments of vSMCs and dynamically responsive to redox status. Intense cytoplasmic and perinuclear staining, coupled with enhanced nuclear localization, was observed in vSMCs, but not HepG2 cells, treated with benzo(a)pyrene (BaP), H_2_O_2_, or N-acetylcysteine, agents known to modulate redox status. 3′ RACE indicated that p70 is not generated as a product of endogenous gene expression, but rather taken up from the extracellular compartment. While p70 was undetectable in cells grown for 24 hours under serum-free conditions, cell-associated, acid-resistant albumin was detected 30 min after the addition of exogenous albumin. vSMCs incubated at 4°C with 100 **μ**g/mL unlabeled BSA and 10 **μ**g/mL FITC-BSA for 60 minutes and switched to 37°C to examine temperature-sensitive label uptake showed punctate structures throughout the cell consistent with albumin internalization at the higher temperature. Albumin was found to influence redox-signaling, as evidenced by modulation of cyp1a1 gsta1 and Ha-ras gene inducibility. Together, these results implicate albumin and albumin-like proteins as critical regulators of vascular redox signaling.

## 1. Introduction

Aerobic organisms are continually subjected to endogenous and exogenous stressors that regulate redox homeostasis. As part of the adaptive response to oxidative insults, aerobes have evolved a variety of proteins that sense changes in redox homeostasis and convey signals to the nucleus to regulate gene expression. One prominent redox sensing mechanism involves the activation of a *cis*-acting regulatory element known as the antioxidant response element (ARE). ARE sequences are present in the 5′ UTR of genes involved in redox homeostasis [1, 2], growth regulation [3, 4], and drug metabolism [5, 6]. The transcriptional response mediated through AREs is complex and varies according to cell type, gene context, and chemical specificity [7]. AREs are regulated by polycyclic aromatic hydrocarbons, such as benzo(a)pyrene (BaP), phenolic antioxidants such as tert-butyl hydroquinone (tBHQ), or direct oxidants, such as H_2_O_2_ [7]. 

Redox signaling is of utmost importance in vascular cells where reactive oxygen species function as signaling molecules regulating important aspects of vascular physiology and where tight control of oxidative metabolism is essential for the preservation of structural and functional integrity. Multiple signal transduction cascades including NF-*κ*B, ERK1/2, AP-1, and ARE have been characterized as critical elements of regulatory control within the vascular wall. vSMCs play a central role in the regulation of vascular redox signaling as evidenced by their involvement in the regulation of matrix metalloproteinases, paracrine regulation of endothelial function, and expression of proinflammatory molecules with strong atherogenic effects. Therefore, the study of vascular redox signaling is essential to advancements in our understanding of molecular vascular cell biology and modalities for therapeutic intervention to effectively prevent atherogenesis.

In efforts to characterize the molecular response to oxidative stress in vascular smooth muscle cells (vSMCs), we have focused on characterization of the multiprotein complex assembled on redox-regulated genes. Several ARE interacting proteins have been identified in vSMCs, including the Cap“n”Collar (CNC) proteins, Nrf1 and Nrf2, and c-Jun, and aryl hydrocarbon receptor (AHR), small Maf proteins, and ΦAP3 [8]. ARE binding proteins belong to the leucine zipper family of transcription factors and dimerize with similar or related proteins to mediate gene activation or repression. Itoh et al. [9] first described a repression mechanism in response to electrophilic stress involving Nrf2 and Kelch-associated protein-1 (Keap-1). Addition of prooxidants overcomes this negative interaction and activates ARE-regulated transcription. Keap-1 is a homologue of the *Drosophila* actin-binding protein Kelch, a relationship consistent with the interactions of Nrf2 and Keap-1 with the actin framework in response to redox stress. 

A p70 albumin-like protein was identified as a novel component of the redox sensing protein machinery of vSMCs [8]. BLAST analysis of this protein established homology to Bach2 and to several zinc-finger proteins containing homeodomains. Three domains of 100% homology are shared between albumin-like proteins and Bach2, suggesting that these structural features may be relevant to redox signaling. Bach2 possesses CNC and Broad-Complex, Tramtrack, and Bric-a-Brac (BTB) domains known to be critical for interaction with ARE sequences [10]. The BTB motif is also involved in regulation of Nrf2 interactions with other transcriptional regulatory proteins, such as N-CoR and SMART [11]. Bach2 is known to associate with Maf proteins via the CNC motif to participate in transcriptional control [12]. BTB proteins often contain Kelch domains that mediate interactions with the cytoskeleton; a relationship consistent with our previous finding that actin participates in ARE signaling [8]. Hoshino et al. [13] have suggested that oxidative stress abolishes nuclear export of Bach2, thus, implicating nuclear accumulation of this protein in a redox signaling. The involvement of albumin-like proteins in transcriptional control is best exemplified by the role of vitamin D binding protein (DBP) in sterol binding and activation of vitamin-D-regulated transcription [14]. Of special note is the finding that albumin and DBP share homologous patterns of disulfide bridge formation and protein folding [15]. 

On the basis of the above findings we hypothesized that p70 is involved in redox signaling in vSMCs. Evidence is presented here that p70 is dynamically regulated by oxidative stress and that its intracellular localization is dependent upon extracellular protein uptake. The translocation of p70 from the cytosolic to nuclear compartment is involved in redox regulation of vascular gene expression.

## 2. Materials and Methods

Cell culture and chemical treatment: cultures of C57/BL6 mouse aortic vSMCs were grown in Media 199, HepG2, and HEK293 cells in Eagle's Minimum Essential Medium and COS7 in RPMI 1640 supplemented with 10% fetal bovine serum (FBS). For chemical treatments, cells were challenged with either BaP or H_2_O_2_ at 37°C and 5% CO_2_ for various times and concentrations as noted. In studies to determine if albumin is taken up from the extracellular medium, cells were incubated in serum-free ExCell 293 medium to deplete endogenous albumin-like protein stores.

Protein extraction: cultures were rinsed twice with prewarmed PBS and harvested by scraping plates with Buffer A (20 mM HEPES—pH 7.6; 1.5 mM MgCl_2_; 0.2 mM EDTA; 10% glycerol; 0.5 mM DTT; Complete Mini (Roche) protease inhibitor cocktail) and placed on ice for 10 minutes. Cells were dounce-homogenized and centrifuged at 14,000 × g for 20 minutes and supernatant (cytosol) collected and stored at −80°C. The nuclear pellet was redissolved in buffer B (20 mM HEPES—pH 7.6; 420 mM NaCl; 1.5 mM MgCl_2_; 0.2 mM EDTA; 25% glycerol; 0.5 mM DTT; Complete Mini (Roche) protease inhibitor cocktail), incubated on ice for 1 hour, and supernatant collected after centrifugation at 14,000 × g for 20 minutes. 

Anti-p70 polyclonal antibody production: female rabbits were purchased from Harlen Laboratories and immunized with a KLH-conjugated 17-mer peptide corresponding to the N-terminus of albumin-like protein. The sequence used for immunization corresponds to the N-terminus of the albumin-like protein identified previously [8] and extended based on resequencing of the originally described 12-mer peptide. After sufficient amounts immunoglobulin was detected by ELISA titers, animals were bled and crude polyclonal serum collected.

Western analysis and immunofluorescence microscopy: for western analysis, cytosolic and nuclear protein extracts were electrophoresed on 4–12% gradient polyacrylamide gels as described previously [8]. Immunofluorescence detection was carried out as described in [16]. Briefly, vSMCs were seeded at a density 43 cells/mm^2^ in 10 cm dishes containing super frost/plus microscope slides (Fisher). Cultures were preincubated for 1 hr with N-acetyl-cysteine (0.5 mM) prior to challenge with 0.3 or 3 *μ*M BaP or vehicle for 3 hours. Cells were fixed in cold acetone/methanol and p70 was visualized by immunofluorescence. Nuclei were counterstained with Dapi or phalloidin 568 (Molecular Probes). Cover slips were mounted using Prolong Antifade (Molecular Probes) and slides were kept in chambers with desiccant at 4°C. Twenty images per slide per antibody were captured from ~400 cells in triplicate experiments. Images were filtered based on intensity and size to quantify positive events using the Zeiss Axiovision Rel 4.3 image analysis software and processed for analyses.

p70 cloning: 3′ RACE approach was used to clone p70 cDNA. Total RNA was isolated from vSMCs, mouse or rat liver or 11-day mouse embryos using TRIzol (Invitrogen). RNA samples were treated with DNAse I (DNA-free kit, Ambion) and 0.25 *μ*g RNA reverse transcribed in 20 *μ*L with SuperScript II (Invitrogen) using an oligo (dT17) anchored with specific oligo GACTCGAGTCGACATCGA(dT17). The products of the reverse transcription reaction (2 *μ*L) were amplified using 3′-PCR primers, GACTCGAGTCGACATCGA and specific primers designed against the amino-terminal coding sequence of mice (CGCCCATCGGTATAATGAT), rat (GCCCATCGGTTTAAGGAC), or mice and rat (GAAGCACACAAGAGTGAG) albumin. In complementary experiments, 15 degenerate oligonucleotides were designed to amplify the p70 amino-terminal coding sequence (primer sequences available upon request) and annealed at 49–51°C for 15 sec, depending on the primer, extended at 68°C (1 kb/min), and subjected to variable numbers of cycles following denaturation at 94°C (15 sec). 

Albumin uptake assays: the assays were essentially done as described by Siddiqui et al., 2004 [17]. Briefly, vSMCs were grown to subconfluence on either glass cover slips in 6-well culture plates or culture slide chambers in DMEM with 10% FBS. Prior to the assay, cells were grown for 24 hours in ExCell 293 medium to starve for albumin. The cells were washed with either cold growth medium or PBS in some experiments and incubated with ExCell 293 made with 100 *μ*g/mL unlabeled BSA and 10 *μ*g/mL FITC-BSA for 60 minutes at 4°C for the binding. It is well established that at this temperature albumin internalization does not occur. After 60 min plates were placed in the CO_2_ incubator at 37°C incubator and at different time points, plates were removed, put on ice, and washed with ice-cold PBS; 100 mM Na-Ac, pH 2.5, and 150 mM NaCl (to remove cell surface albumin); and with 100 mM Na-phosphate at pH 7.4 (to restore the pH to neutral). The antifade agent in glycerol 5% was added to the mounting medium to reduce photobleaching. Fluorescence of cells was monitored using FITC filter to visualize the internalized albumin. DAPI staining was used to stain nuclear and mitochondrial DNA. Images were visualized using a Zeiss Axiovert inverted fluorescence microscope.

*Statistical Analysis*. The significance of findings was calculated at the *P* < 0.05 using Students *t*-test or nonparametric Wilcoxon rank test.

## 3. Results

### 3.1. Compartmentalization of p70 in vSMCs

**Figure 1:**
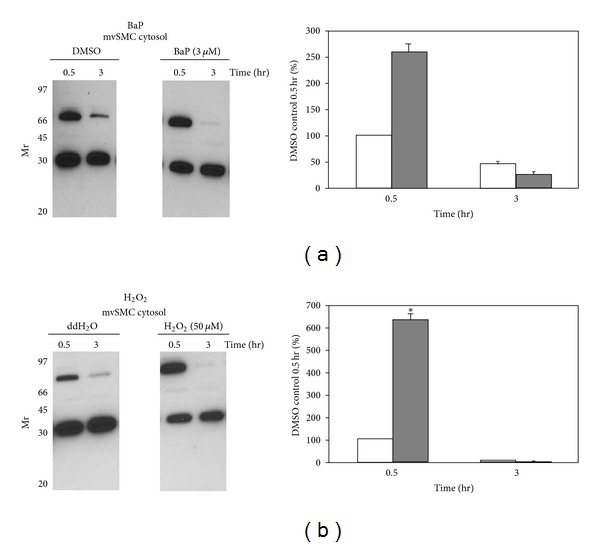
p70 expression in the cytosolic compartment of vascular smooth muscle cells—time-course and oxidant treatment. Panel (a) shows that mouse vSMCs were treated with 3 *μ*M BaP or vehicle (DMSO) for 0.5 or 3 hr. Duplicate cell samples were processed for reproducibility. Panel (b) shows vSMCs were treated with 50 *μ*M H_2_O_2_ or vehicle (ddH_2_O) for 0.5 and 3 hr. p70 expression was normalized against p35, a cellular protein recognized specifically by the antibody but not involved in the cellular response to oxidative stress. Densitometric values denote the average of duplicate samples from 3 independent experiments.

**Figure 2:**
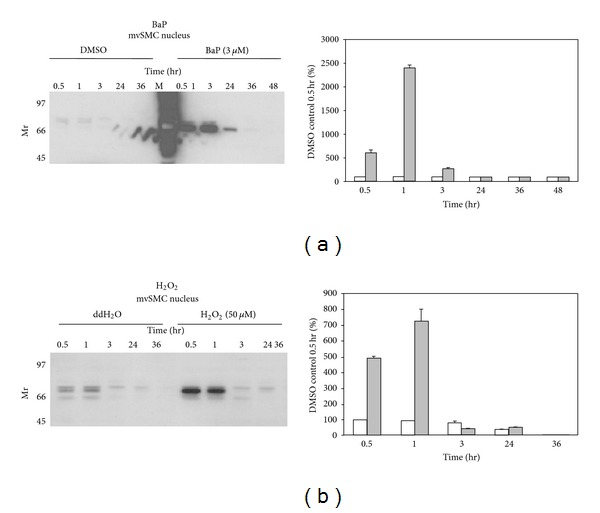
p70 Expression in the nuclear compartment of vascular smooth muscle cells—time-course and oxidant treatment. Panel (a) shows that vSMCs were treated with 3 *μ*M BaP or vehicle (DMSO) for 0.5–48 hr. Panel (b) shows vSMCs were treated with 50 *μ*M H_2_O_2_ or vehicle (ddH_2_O) for 0.5–36 hr as above. Densitometric values denote the average for each time and treatment in 3 independent experiments.

**Figure 3:**
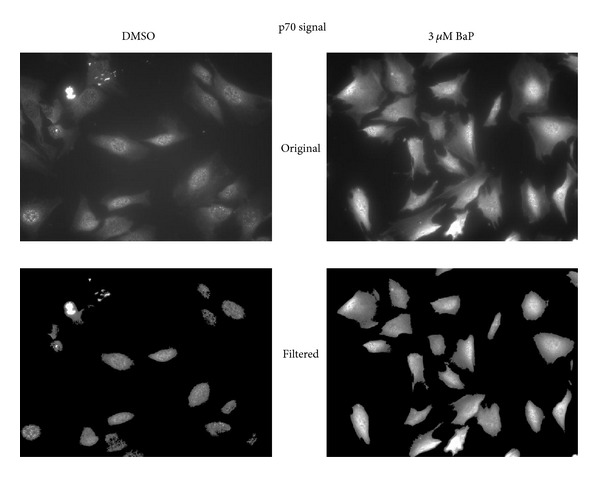
Immunofluorescence localization of p70 in vascular smooth muscle cells. vSMCs were treated with vehicle or 0.3 or 3 *μ*M BaP for 3 hr and then processed for immunofluorescence detection as described in Section 2. Panel (a)—morphometric analysis of total immunofluorescence signal before and after filtration for cellular signal. Panel (b)—morphometric analysis of total immunofluorescence signal before and after filtration for nuclear signal. Panel (c)—the interaction with N-acetylcysteine was examined in experiments where cultures were pretreated with 0.5 mM N-acetylcysteine and then challenged with 0.3 or 3 *μ*M BaP or DMSO. Similar results were seen in 3 independent experiments.

Female rabbits were immunized with a KLH-conjugated 17-mer peptide corresponding to the N-terminus of p70 to generate an affinity purified polyclonal antibody. A 70-kDa protein was detected in cytosolic extracts of vSMCs and this response was enhanced in cells challenged with chemical oxidants (Figures 1(a) and 1(b)). Cytosolic p70 signal decreased as a function of time under both control and oxidant-stimulated conditions, suggesting that the stability and/or intracellular localization of this protein is subject to regulation in vSMCs. A 30 kDa immunoreactive protein was also detected by the p70 antibody, but signal intensity remained unchanged as a function of time or chemical treatment indicating that this protein is not likely involved in redox signaling. The specificity of the antibody was confirmed in studies showing that incubation of anti-p70 antiserum with 1, 5, or 10 *μ*g p70 17-mer peptide completely neutralized the immunoreactive signal (not shown). p70 was selectively enriched in the nucleus of BaP- or peroxide-treated vSMCs (Figures 2(a) and 2(b)), indicating that redox stress regulates intracellular localization of this protein. Nuclear detection was seen shortly after oxidant treatment, as evidenced by marked signal increases as early as 0.5 hr that lasted for 1–3 hr. Regulation of p70 compartmentalization exhibited cell-type specificity since cytosolic or nuclear expression was unchanged in HepG2 cells treated with BaP (not shown). To further evaluate the compartmentalization of p70, immunofluorescence measurements were carried out in cells challenged with oxidants in the presence or absence of N-acetylcysteine, a modulator of redox status in vSMCs [18]. Cytoplasmic and perinuclear staining was observed under basal conditions in vSMCs (Figures 3(a) and 3(b)). As reported earlier, treatment with BaP (0.3 or 3 *μ*M) for 3 hr enhanced nuclear localization of p70 (Figure 3(c)), while pretreatment with 0.5 mM N-acetylcysteine enhanced nuclear localization of p70 and selectively neutralized the response elicited by 3 *μ*M BaP. Collectively, these findings confirm that modulation of redox homeostasis by cellular oxidants and antioxidants modulates intracellular compartmentalization of p70 and induces transient nuclear localization in vSMCs. 

### 3.2. p70 Cloning Efforts

**Figure 4:**
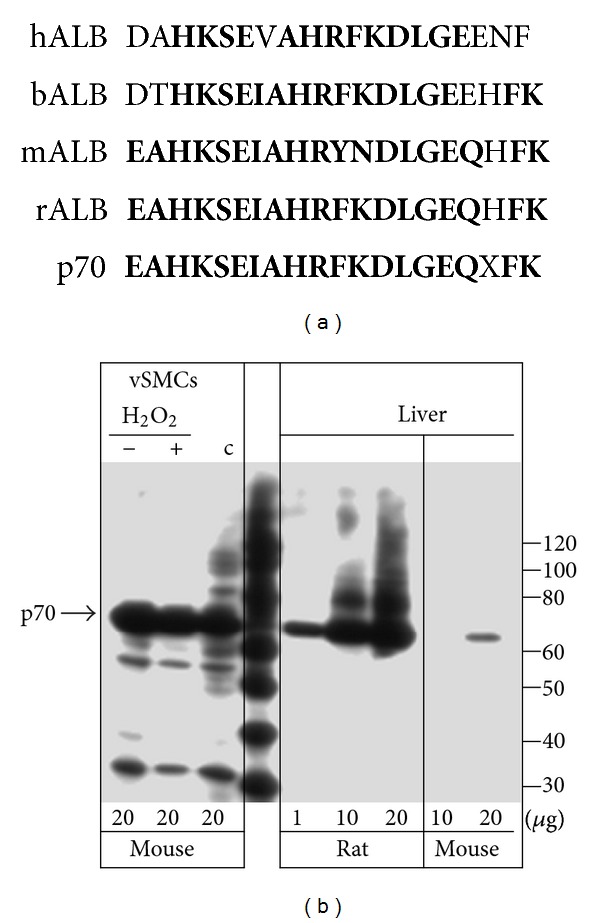
Structural correlates of p70. Panel (a)—Comparison of amino-termini (amino acids 25–44) sequences in different albumin proteins and p70. hALB, human; bALB, bovine; mALB, mouse; rALB, rat albumin. X, unidentified amino acid in p70. Conserved amino acids are in bold. Panel (b)—affinity of p70 antibody to proteins in cell extracts obtained from mouse vSMCs or rat and mouse livers. Different amounts of extracts were fractionated and probed with p70 antibody to ascertain relative protein abundance. Similar results were seen in 2 independent experiments.

The N-terminal 17 amino acid sequence of p70 is different from that of mouse albumin by two amino acids, but identical to rat albumin (Figure 4(a)). Therefore, experiments were conducted to determine if the p70 antibody recognizes mouse and/or rat albumin. Total protein was isolated from mouse vSMCs, rat or mouse liver extracts, and processed for western analysis. The p70 antibody had considerably lower affinity to mouse albumin compared to rat (Figure 4(b)), consistent with differences in the amino acid composition of the peptide sequence between mouse albumin and p70. Surprisingly, signal intensity of p70 in vSMCs extracts was comparable to that from rat liver, suggesting that p70 is different from mouse albumin. On the basis of these findings, we set out to determine if p70 is expressed *in vivo* or is limited to *in vitro* growth of cells in culture. A 3′ RACE approach was used to clone p70 cDNA. Considering the extensive amino acid sequence homology between mouse and rat albumin mRNAs, two specific oligonucleotides were designed to amplify a region homologous to both species. cDNAs were obtained from total RNA isolated from vSMCs, or rat and mouse liver using oligo(dT) anchored with specific oligonucleotide. In the second round of PCR, we used specific oligonucleotides and the anchor oligonucleotide to prime second strand DNA synthesis of all cDNAs obtained from RNAs with poly(A) tail. A specific PCR product was obtained from RNA isolated from rat liver, which corresponded to rat albumin, as determined by sequencing (data not shown). However, no specific product was amplified using RNA from vSMCs. In contrast, we obtained albumin cDNA from both rat and mouse with oligonucleotide recognizing both mRNAs. These controls indicate high specificity of the RT-PCR assay. No specific product was amplified using either oligonucleotide with two different RNA preparations from vSMCs. Although any other gene known to be specifically expressed in vSMCs, such as calponin and vSMC *α*-actin, was amplified (data not shown). Therefore, we speculated that p70 codon usage might be different from that of rat and mouse albumin. To address this question, we designed 15 different degenerate oligonucleotides according to the coding capacity of p70 N-terminus and repeated the PCR. There was no specific product amplified with different RNA preparations from either vSMCs or 11-day mouse embryo (data not shown). We used RNA isolated from mouse embryos, because expression of p70, although low, can be detected by western analysis (data not shown). Therefore, we concluded that p70 is not a product of endogenous gene expression in vSMCs.

### 3.3. Cellular Uptake of p70

**Figure 5:**
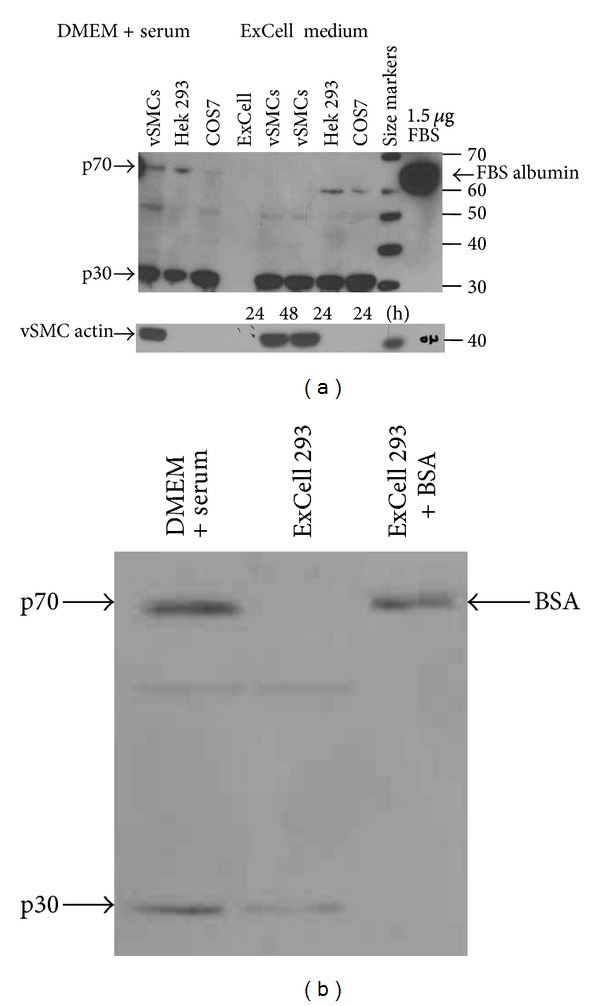
Cellular expression of p70. Panel (a)—p70 is not detected in vascular smooth muscle cells, HEK293, or Cos-7 cells after growth in serum-free medium. vSMCs, HEK293, and COS7 cells were grown either in the presence of serum (DMEM plus serum) or in serum-free medium (ExCell) and cellular extracts analyzed by western blotting using p70 antibody. FBS (extreme right lane) served as a positive control. Panel (b)—detection of p70 and BSA in vascular smooth muscle cells. Cell-associated albumin can be detected with p70 antibodies upon albumin addition for 30 min. Similar results were seen in 2 independent experiments.

The amino acid sequence of p70 matches cleaved albumin at its amino terminus suggesting that p70 is taken up by cells following appropriate signals. To determine if vSMCs internalize albumin present in the growth medium, vSMCs were grown in the absence or presence of serum in ExCell 293 medium and processed for Western analysis.  Figure 5(a) demonstrates that p70 immunoreactivity is not detected in ExCell medium for 24 h, in different cell lines (vSMCs, Hek 293, and COS7), but is detected when cells are grown in medium supplemented with FBS. The level of p70 in COS7 cells is low compared to other cell types. It has been demonstrated that uptake of albumin by vascular endothelial and lung epithelial cells is mediated by TGF-*β* receptor, and COS7 cells express neither TGF-*β*I nor TGF-*β*II receptors and therefore have compromised uptake of exogenous albumin [17]. The observation that COS7 cells have low levels of p70 suggests that vSMCs and HEK 293 cells internalize p70 from the medium, and that this internalization may be dependent on TGF-*β* receptors. Of interest was the finding that p30 immunoreactivity was retained under conditions where extracellular albumin uptake from the extracellular medium was inhibited.

**Figure 6:**
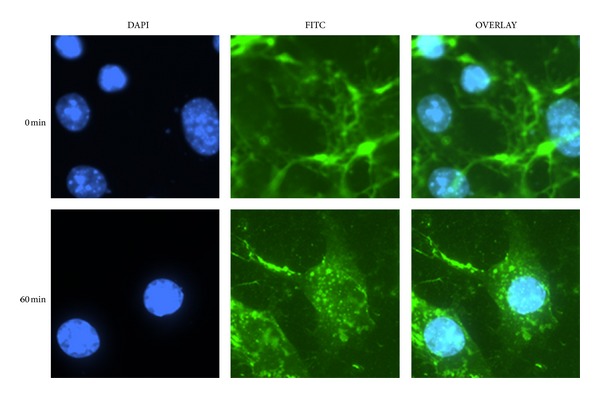
Albumin uptake in vascular smooth muscle cells. Cell-internalized FITC albumin was visualized after removing the external FITC-albumin as described in Section 2. Cell accumulated albumin shows punctate staining. DAPI staining denotes the nucleus. Similar results were seen in 3 different experiments.

Next, we sought to determine if external albumin is internalized by vSMCs. Cells were incubated for 24 hr in serum-free ExCell 293 medium to starve the cells for albumin and then switched to ExCell 293 medium containing 2.5 mg/mL BSA, the amount of albumin present in the medium when cells grown are grown in the presence of 10% FBS.  Figure 5(b) demonstrates that p70 is not detectable in vSMCs grown under serum-free conditions for 24 hr. Following acidic washes to remove nonspecifically bound albumin, cell-associated albumin was detected as early as 30 min after addition of albumin to the medium. FITC-labeled albumin was then used to distinguish between surface bound and internalized albumin.  Figure 6 shows specific surface association of extracellular albumin when cells are incubated with FITC albumin for 1 hr at 4°C. The fluorescence pattern shifted to an intracellular punctuate pattern upon incubation of the cells at 37°C, indicating that a temperature-sensitive mechanism participates in compartmentalization of cellular albumin. Together, these findings indicate that surface-associated albumin is actively internalized by vSMCs and that regulatory mechanisms exist that modulate albumin mobilization across cellular membranes.

**Figure 7:**
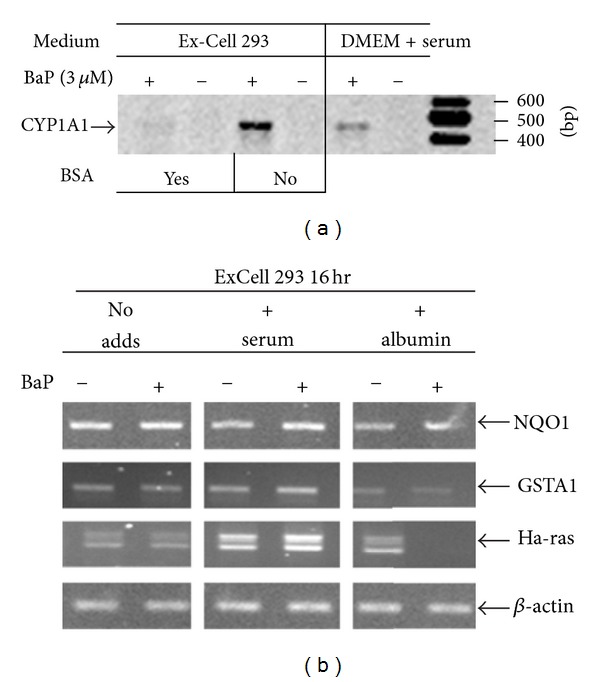
Regulation of gene inducibility in vascular smooth muscle by albumin. vSMCs were grown in the presence of serum (DMEM plus serum), or in serum-free medium (ExCell 293). Panel (a) shows patterns of cyp1a1 inducibility by BaP in the presence or absence of extracellular albumin, ExCell 293 was supplemented with BSA (100 *μ*g/mL) for and then challenged with BaP (3 *μ*M) or vehicle (DMSO) for 24 hr. Total RNA was analyzed for cyp1a1 gene expression by RT-PCR and gel-electrophoresis. DNA ladder in basepairs (bp) was loaded at the extreme right. Similar profiles were seen in 4 independent experiments. Panel (b) shows patterns of redox regulated expression for NQO1, GSTA1 and Ha-ras in the presence or absence of extracellular albumin and BaP. Samples were processed as described above. Similar profiles were seen in 3 independent experiments.

To examine the involvement of p70 in redox signaling, we compared the inducibility of cyp1a1 by BaP in vSMCs grown in serum-free medium before and after addition of bovine albumin (Figure 7(a)). Cyp1a1 is a member of the large superfamily of cytochromes P450 (CYPs) encoding for heme-containing monooxygenases involved in the hydroxylation of steroids and xenobiotics [18]. This gene is not expressed under constitutive conditions, but markedly induced by ligands of the aryl hydrocarbon receptor (AHR), a basic helix-loop-helix transcription factor involved in stress signaling in vSMCs. Reciprocal interactions between AHR and redox signaling have been documented, such that activation of the redox machinery inhibits cyp1a1 inducibility via oxidative modification of cysteine residues within the transactivation domain of redox-regulated transcriptional regulators [18, 19]. Thus, our approach enabled us to simultaneously examine the integrity of AHR and redox signaling as a function of albumin uptake. The expression of cyp1a1 in vSMCs was determined by RT-PCR before and after addition of BSA (2.5 mg/mL), a concentration that is representative of that achieved with 10% FBS. As expected, no cyp1a1 expression was detected in the absence of inducer under any experimental condition examined (Figure 7(a)). BaP challenge induced cyp1a1 in the presence or absence of serum, but gene induction was markedly enhanced in the absence of albumin. Next, we evaluated the inducibility profiles of nqo1, gsta1, and H-ras, vSMC genes known to be regulated via redox signaling through the activation/repression of AREs [3, 7, 8, 18]. No changes in the constitutive or inducible expression of nqo1 were observed in the presence or absence of albumin. In marked contrast, albumin repressed constitutive expression of gsta1 and failed to support inducibility of the gsta1 gene by 3 *μ*M BaP. For Ha-ras, albumin inhibited both constitutive and inducible expression of the gene. These findings are consistent with the involvement of albumin in the assembly of macromolecular complexes that interact specifically with AREs in the regulatory region of target genes [8]. Together, these findings implicate a dynamic system in vSMCs responsible for intracellular localization of albumin under conditions of oxidative stress, and the association of albumin with transcription factors that modulate the adaptive cellular responses to oxidative stress. 

## 4. Discussion

p70 was first identified as an albumin-like protein within the macromolecular protein complex assembled on the AREs of redox-regulated genes [8]. A role for p70 as a signal transducer is consistent with the ability of other albumin-like proteins, such as DBP to bind sterols to convey transcriptional signals to the nucleus [14]. DBP is positioned on chromosome 4 in close proximity to chemokines and is believed to participate in redox-regulated inflammatory signaling [20]. Interestingly, a 70 kDa albumin-like protein has been described in the cornea that is modulated by oxidative stress [21]. BLAST analysis of p70 has shown homology to Bach2, a protein containing CNC and BTB domains that participate in the regulation of ARE signaling [10, 14, 22]. Cantin et al., 2000 [23], have shown that albumin modulates NF-*κ*B activation and cellular GSH levels. Furthermore, serum albumin has been shown to modulate vSMC energy metabolism in carotid artery strips where extracellular albumin is taken up and broken down into byproducts that stimulate oxygen consumption and augment glucose oxidation [24]. 

An earlier report identified an albumin-like protein as the melanoma associated antigen, B700. B700-like molecules are produced by melanomas of murine, human, swine, and hamster origin [25–33]. The primary structure and biochemical functions of B700, as well as its *in vivo* metabolic fate, are largely unknown. B700 can be detected on the cell surface as well as the intracellular compartment in various murine cell lines [29]. Despite a significant homology to the mammalian serum albumins, monoclonal antibodies produced by eight different rat hybridoma cell lines against the melanoma-specific B700 antigen cross-react only with murine albumin [27]. The fact that both murine and human melanoma cells produce B700 antigen which is recognized by murine albumin antibodies suggests the presence of a murine-type albumin in human melanoma. Surprisingly, BLAST search retrieved a human clone which is completely homologous to the rat albumin (accession number AX430341). To date, no linkage between p70 and B700 has been established. Our inability to clone a mouse albumin-like protein in vSMCs using 3′ RACE suggests that either a unique gene product is not expressed in vSMCs or that an mRNA encoding p70 lacks a conventional polyA tail. The results of our studies, however, indicate that a more likely possibility is that extracellular albumin as well as other albumin-like proteins are taken up by vSMCs from the extracellular compartment and engaged in intracellular signaling. Within this context, previous studies have shown that colon, kidney, and lung epithelial cell, as well as vascular endothelial cells take up albumin [34]. Acute exposure of human proximal tubular epithelial cells to albumin induces IL-8 gene and protein expression in a time and dose-dependent manner and stimulates IL-8 secretion [34]. These investigators also demonstrated translocation of NF-*κ*B into the nucleus after exposure to human albumin, as well as enhanced production of reactive oxygen species. 

The nuclear localization of p70 following oxidative stress coupled to the modulation of redox-sensitive gene expression strongly implicates albumin as a key participant in redox signaling in vSMCs. Although a 30 kDa protein was consistently detected in vSMCs, the localization of this protein was not dependent upon chemical oxidant treatment, suggesting that its involvement in p70 redox biology is unlikely. Albumin acts as a powerful antioxidant in cell culture [35], and is known to bind, sequester and stabilize a variety of unstable molecular species [36]. This acidic, soluble protein has both high-affinity and secondary binding sites and optimizes the roles that fatty acids, metals, disulfides, and other molecules in cellular metabolism [37–40]. Vascular endothelial cells undergo albumin endocytosis using TGF-*β* receptor, and activation of the receptor stimulates endocytosis which results in TGF-*β*RII signaling leading to Smad2 phosphorylation and Smad4 translocation to the nucleus [17]. In addition, albumin endocytosis induces phosphorylation of p38 MAPK resulting in activation of endothelial proliferation and reduction of endothelial apoptosis [41]. 

## 5. Conclusions and Clinical Implications

This study implicates albumin and albumin-like proteins in the regulation of redox signaling in vSMCs. These proteins are present in the macromolecular complex assembled within the 5′-regulatory region of redox-sensitive genes and appear to be dynamically responsive to changes in redox status following internalization into vSMCs from the extracellular compartment via temperature-sensitive transport mechanisms. Future studies are required to determine if the ability of albumin and albumin-like proteins to repress ARE signaling involves solely regulation of macromolecular protein assembly during transactivation of redox-regulated genes, or redox sensing via molecular interactions between cysteine residues and chemical electrophiles. Collectively, these findings raise important questions about the cellular and molecular mechanisms responsible for the clinical effectiveness of albumin, not only in the restoration of oncotic pressure, but also in the management of patients with hypovolemia, shock, burns, surgery or trauma, cardiopulmonary bypass, acute respiratory distress syndrome, and hemodialysis. The present study suggests that the antioxidant mechanism of these proteins may warrant further investigation.
